# Microencapsulated Neural Stem Cells Inhibit Sciatic Nerve Injury-Induced Pain by Reducing P2 × 4 Receptor Expression

**DOI:** 10.3389/fcell.2021.656780

**Published:** 2021-09-21

**Authors:** Wen-jun Zhang, Chen Luo, Chao Huang, Si-cheng Liu, Hong-liang Luo

**Affiliations:** The Second Affiliated Hospital, Nanchang University, Nanchang, China

**Keywords:** neural stem cells, neuropathic pain, P2 × 4 receptors, chronic sciatic nerve compression injury, microencapsulation

## Abstract

**Objectives:** The purpose of this study is to investigate the effects of transplantation of microencapsulated neural stem cells (MC-NSCs), which downregulate the P2 × 4 receptor (P2 × 4R) overexpression and relieve neuropathic pain (NPP).

**Methods:** Neural stem cells (NSCs) and MC-NSCs were transplanted to the injured sciatic nerve. Transmission electron microscope and immunofluorescence were used to observe the changes of injured sciatic nerve. Behavioral methods were used to detect mechanical withdrawal thresholds (MWT) and thermal withdrawal latency (TWL) of rats. Expression levels of P2 × 4Rs and p-p65 in the spinal cord segment of rats were measured by using molecular biology methods. The concentrations of IL-1β and TNF-α were detected in serum of rats by ELISA.

**Results:** After sciatic nerve injury, the sciatic nerve fibers had the myelinated lamina separated, and disintegrated fragments could be seen. The fluorescence intensity of myelin MBP was weakened. The MWT and TWL were significantly decreased, the expression of P2 × 4Rs and p-p65 were significantly increased, and the concentrations of IL-1β and TNF-α were increased. After NSC and MC-NSC transplantation, the myelin sheath of the sciatic nerve was relatively intact, some demyelination changes could be seen, and the injured sciatic nerve has been improved. The fluorescence intensity of myelin MBP was increased. The MWT and TWL were increased, expression levels of P2 × 4Rs and p-p65 were decreased, and the concentrations of IL-1β and TNF-α were significantly decreased. Compared with NSC transplantation, transplantation of MC-NSCs could better repair the damaged sciatic nerve, decrease the expression of P2 × 4Rs and p-p65, decrease the level of IL-1β and TNF-α, and relieve pain (all *p*-values < 0.05).

**Conclusion:** NSCs and MC-NSCs transplantation may alleviate pain by reducing the expression of P2 × 4Rs and inhibiting the activation of NF-KB signaling, while MC-NSCs transplantation has a better effect of suppressing pain. Our experimental results provide new data support for the treatment of NPP.

## Introduction

Neuropathic pain (NPP) is a common symptom of most diseases in the clinic, and its pathological mechanism is very complex, but there is no good treatment method, which seriously affects the quality of life of patients (Seixas et al., 2014). Clinically, drugs are usually used to suppress pain, but the treatment effect is not good (Green-Fulgham et al., 2019). Therefore, exploring and researching the treatment method of NPP has attracted the interest of most researchers. Numerous studies have shown that P2 × 4Rs play an important role in the development of NPP (Williams et al., 2019). After nerve injury, P2 × 4Rs are highly expressed, which can induce and aggravate pain (Yang et al., 2019), while reducing the expression level of P2 × 4Rs can inhibit NPP (Masuda et al., 2014). With the continuous exploration of treatment methods in NPP, cell transplantation technology has entered the field of vision of researchers. Cell transplantation has become a promising method for the treatment of nerve injury and pain (Singh and McGuirk, 2016). The transplantation of cells into the site of the injured nerve can exert continuous analgesic pharmacological effect (Gómez et al., 2018). NSCs are the mother cells in the nervous system, which can continuously update and maintain their own numbers, and secrete a variety of neurotrophic factors (such as neurotrophin Y and vascular growth factor), change the surrounding microenvironment of nerve injury, and repair damaged nerves (Ostermann et al., 2019; Yuan et al., 2019). So, NSCs have great prospects in the treatment of nerve injury. However, transplantation of cells into the host can lead to immune rejection, damage the transplanted cells, and reduce their survival rate. Microcapsule is a kind of translucent biological lipid membrane with immunoisolation and good biocompatibility, which can reduce the immune rejection and damage factor attack of transplanted cells, improve the survival rate, and better repair damaged nerves (Li et al., 2014; Zhang et al., 2018b). However, the study of microencapsulated neural stem cells (MC-NSCs) in the treatment of pain has not been reported. Thus, in this study, we transplanted NSCs and MC-NSCs to the injured sciatic nerve and explored the effects of NSCs and MC-NSCs on P2 × 4 receptor-mediated NPP.

## Materials and Methods

### Experimental Animals

The 144 healthy SD rats (72 males and 72 females, weighing 150–180 g) were provided by the Animal House of Nanchang University. All protocols were approved by the Animal Care and Ethics Committee, China.

### Culture of Neural Stem Cells

Two-week-old pregnant rats were sacrificed, and the uterus was removed and placed in ice-cold PBS. Subsequently, the fetal rat was decapitated, and the skull was separated with a pair of ophthalmic scissors. The meninges and vascular tissues were fully separated under a microscope (Danmic Global, LLC, San Jose, CA, United States), and then the brain tissue was removed and washed three times with PBS. The brain tissue was cut to a size of 1 mm, digested with 5 ml 1.2% trypsin (Doctor DE Biological, Wuhan, China) for 15 min, and then digestion was terminated with DMEM/F12 medium containing 10% FBS (Doctor DE Biological, Wuhan, China), and centrifuged at 1,000 r/min for 5 min. DNA enzyme, 250 μl, 0.5 mg/L (Doctor DE Biological, Wuhan, China), was added and incubated for about 3 min at 37°C, and then centrifuged at 1,000 r/min for 5 min. The supernatant was discarded, serum-free medium containing 20 ng/ml basic fibroblast growth factor, 20 ng/ml epidermal growth factor, 2% B27, and 1% N2 (all from Abcam, Cambridge, United Kingdom) were added [double-antibiotic concentration was 100,000 U/L penicillin (Sigma-Aldrich) and 10,000 U/L streptomycin] and mixed gently. The culture bottle at a density of 2 × 10^8^/L^–1^ was inoculated and placed in a CO_2_ incubator (Antai Air Technology Co., Ltd., Suzhou, China) for incubation (37.5°C, 5% CO_2_). Around a week, neurospheres could be seen to grow out. The mouse anti-nestin (Abcam) was used to identify the purity of NSCs. The results indicated that more than 90% of the cells were positive for nestin (Du et al., 2019).

### Preparation of Microencapsulated Neural Stem Cells

**FIGURE 1:**
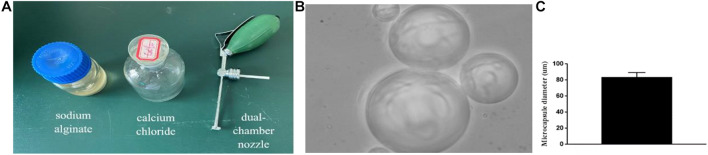
Microencapsulation production: **(A)** Materials and equipment for making microcapsules. **(B,C)** ×400. The produced microcapsule picture and microcapsule diameter histogram.

After identification of the NSCs, we counted the number of cells under a microscope and adjusted the density of the cells to be 2.4 × 10^6^/ml. Then, the NSCs were stained with trypan blue, and the cell survival rate was determined to be greater than 95%. The cell suspension was mixed with 1.5% sodium alginate (Molecular weight 216.121. M to G, 1:4) saline (Boster BioTechnology, Ltd.) at a ratio of 1:1. The mixed solution was sprayed into a small beaker with 20 mmol/L calcium chloride physiological saline mixed solution via a dual-chamber nozzle, and then the unsolidified liquid was poured out after precipitation at room temperature. Physiological saline was used to wash off the excess liquid on the solidified surface two times. Finally, the obtained microcapsules (Figures 1A–C) were re-suspended in salt water for further use or stored in a refrigerator at 4°C (Fannon et al., 2021).

### Establishment of a Model of Injured Sciatic Nerve (Chronic Sciatic Nerve Compression Injury)

The rats in each experimental group were anesthetized by intraperitoneal injection of 2% sodium pentobarbital (Boster BioTechnology, Ltd.). The limbs of the rats were fixed on the operating table, and the skin and subcutaneous tissue of the right hind limb of the rats were opened to expose the sciatic nerve trunk. Subsequently, the middle and upper one-third of the sciatic nerve trunk (distance was 1 mm) was ligated with No. 4 catgut. The strength of ligation should be the twitch of the right hind limb of the rats, so as not to affect the blood flow of the sciatic nerve (Zhang et al., 2018b).

### Transplantation of Neural Stem Cells and Microencapsulated Neural Stem Cells

In the NSC group and MC-NSC group, on the basis of CCI group, the same amount of NSCs or MC-NSCs (2.4 × 10^6^/ml) (Before transplantation of MC-NSCs, trypan blue staining was performed to determine that the cell survival rate was greater than 95%.) were absorbed by a gelatin sponge (Boster Biological Technology, Ltd.) of 1 cm size and then transplanted into the injured side of sciatic nerve. In the sham group, the right hind limb sciatic nerve of the rats was exposed without any treatment. Their right hind limbs were regularly observed (Zhang et al., 2019b).

### Detection of Mechanical Withdrawal Threshold of Rats

The rats were placed in a VonFrey pain threshold measuring glass instrument (Danmic Global, LLC, San Jose, CA, United States) for adaption for 15 min. We used VonFrey filaments to stimulate the plantar of the right hind limb of the rats, and the stimulation intensity gradually increased until withdrawal and paw licking of the rats occurred. Each stimulation time is not less than 15 s. When the right hind limb of the rats was stimulated, the feet of the rats were lifted and retracted, and the minimum stimulation intensity was regarded as the MWT. This was repeated three times, and the average values were taken (Deng et al., 2018).

### Detection of Thermal Withdrawal Latency of the Rats

The BME-410C automatic thermal pain stimulator (Danmic Global, LLC, San Jose, CA, United States) was used to illuminate the plantar of the right hind limb of the rats, and the irradiation time until withdrawal and paw-licking of the rats that occurred were the thermal withdrawal latency (TWL). In order to prevent burns, there should be a 30 s interruption after each irradiation. The experiment was repeated three times, and the average values were taken (Deng et al., 2018).

### *In situ* Hybridization

On days 7 and 14 after operation, the L4–5 spinal cord segment was taken, and the tissues were placed in a frozen slicer (Leica, Bensheim, Germany) for slicing (thickness 10 nm). Three percent citrate pepsin was added for 2 min. The pre-hybridization solution (Boster Biological Technology, Ltd.) was added for 2 h without washing. Then, the hybridization solution (Boster Biological Technology, Ltd.) was added and placed in a 38°C water bath overnight. The next day, the tissues were washed separately in 2 × SSC, 0.5 × SSC, and 0.2 × SSC, respectively, for 15 min. Then, the blocking solution was added for 30 min without washing, biotinylated rat anti-digoxigenin was added for 90 min, and then it was washed with PBS. SABC was added for 20 min, and then it was washed with PBS. Biotin peroxidase was added for 30 min, and then it washed with PBS. Diaminobenzidine color substrate solution (DAB; Boster Biological Technology, Ltd.) was added for 2 min of color development, and then it was rinsed in water. The positive cells of P2 × 4Rs were stained into yellowish-brown and brown. The number of P2 × 4R-positive cells was measured by using Image-Pro Plus 6.0 software (Media Cybernetics, Silver Spring, MD, United States).

The P2 × 4R nucleotide probe sequence is as follows: 5′-TTAACAGTGTGTAGGTGAGGATGGC-3′.

### Western Blotting

On days 7 and 14 after operation, total proteins were extracted from the L4–5 spinal cord segment of the rats. Separating glue and stacking glue separately were prepared. Afterward, the stacking glue was solidified, and the comb was pulled out. A 5-μl molecular marker was added to the first well, and then 10 μl of samples was added to the subsequent wells in the order of the groups, and then electrophoresis and transfer film were performed. After the membrane transfer was completed, the membranes were placed in 5% skimmed milk powder for 90 min. Subsequently, the membranes were placed in the P2 × 4R rabbit polyclonal antibody (1:1,000, Millipore, Bedford, MA, United States), NF-_*K*_B p65 rabbit polyclonal antibody (1:1,000, Sigma-Aldrich), Nf-_*k*_B p-p65 (1:1,000, Sigma-Aldrich), or rabbit β-actin polyclonal antibodies (1:3,000, Boster Biological Technology, Ltd.) and incubated overnight in a 4°C refrigerator. They were placed in goat anti-rabbit secondary antibody (1:5,000, Boster Biological Technology, Ltd.) and incubated for 90 min, and then washed three times with TBST for 10 min each time. Subsequently, the chemiluminescence reaction was carried out. Image Pro Plus version 6.0 image analysis software was used to perform the grayscale analysis of the protein bands. The relative band strength of the target protein was normalized with internal parameters (β-actin).

### RT-qPCR

Total RNA was extracted from the L4–5 spinal cord tissue of rats, and then cDNA was prepared by using FastQuant RT Kit (Tiangen Biotech Co., Beijing, China) with gDNase for PCR amplification (response system: 16°C 30 min, 42°C 30 min, 85°C 5 min). Subsequently, cDNA as a template, and Takara qPCR Kit (Boster Biological Technology, Ltd.) were used to configure a 20-μl system for PCR reaction (reaction system: 95°C 30 s, 95°C 35 s, 60°C 1 min, 95°C 15 s, 32 cycles). Each sample was set with three multiple wells (β-actin was used as internal parameters).

P2 × 4R primers:

F: 5′-CAGATCAAGTGGGACTGCAACC-3′

R: 5′-ACACGATGATGTCAAAGCGGATG-3′

β-actin primers:

F: 5′-TAAAGA CCTCTATGCCAACACAGT-3′

R: 5′-CACGATGGAGGGGCCGGACTCATC-3′

### ELISA

At 7 and 14 days after operation, the concentration levels of IL-1β and TNF-a were measured in serum by using an ELISA kit (Boster Biological Technology, Ltd.). We collected blood from the orbits of rats, and then serums were collected by centrifugation. Subsequently, the test was performed according to the instructions of the ELISA kit.

### Ultrastructural Changes of the Sciatic Nerve

On days 7 and 14 after surgery, the injured sciatic nerve of rats was fixed with 2.5% glutaraldehyde solution, embedded in resin, and made into 10-μm flakes. The slices were stained with osmic acid after Wright’s staining and fixing. Pathological changes in the nerve were observed under transmission electron microscopy (FEI, Hillsboro, OR, United States).

### Immunofluorescence

The tissues were attached to the slide and placed in a wet box. The tissue sections were dewaxed, rehydrated, and then repaired with antigen. The rabbit primary antibodies (P2 × 4Rs 1:100 and MBP 1:100) and goat anti-rabbit secondary antibody (1:200; Biyuntian Biological Technology Co., Ltd., Shanghai, China) were added. DAB chromogenic solution was then added for 2–5 min, and the nuclei were stained with hematoxylin for 1–2 min. The samples were then dehydrated, transparent, and sealed. Staining of the positive cells was observed under an inverted microscope.

### Statistical Analysis

SPSS19.9 software (SPSS; Chicago, IL, United States) was used to perform the statistical analysis. Data were expressed as the mean ± SD, and the experiments were repeated three times. Differences between groups were determined by one-way analysis of variance (ANOVA) followed by Fisher’s *post hoc* tests for multiple comparisons. We used a two-way ANOVA, followed by the *post hoc* test to detect the differences among the groups in the behavior studies. Differences of *p* < 0.05 were considered to be significant.

## Results

### Ultrastructural Changes of Injured Sciatic Nerve and Myelin Basic Protein Staining Changes

**FIGURE 2:**
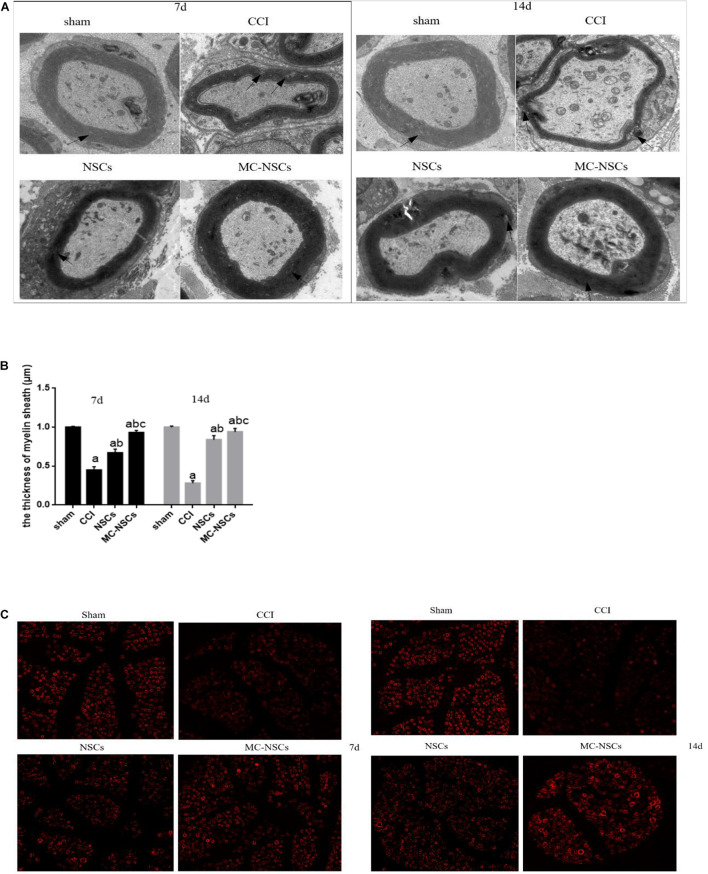
On days 7 and 14 after surgery, the changes in the sciatic nerve myelin ultrastructure and fluorescence in each group of rats were randomly observed. **(A)** Electron microscope at ×5,000 magnification. In the sham group, the structure of the myelin sheath of the sciatic nerve was uniform, regular, and clear. However, in the chronic sciatic nerve compression injury (CCI) group, the sciatic nerve fibers had the myelinated lamina separated, segmental, or stratified demyelination changes could be seen in part of the myelin sheath. In the neural stem cell (NSC) group, the myelin sheath of the sciatic nerve was relatively intact, and some demyelination changes could be seen. Compared with the NSC group, in the microencapsulated neural stem cell (MC-NSC) group, the sciatic nerve had more myelinated fibers, the thickness of the myelin sheath was more uniform, and the morphology of the nerve filaments was normal. **(B)** The thickness of the myelin sheath of the sciatic nerve of each group of rats. Data were expressed as the mean ± SD (*n* = 6). **(C)** At × 400 magnification. Immunofluorescence assay was used to detect the changes in myelin basic protein (MBP) fluorescence in sciatic nerve myelin sheath of rats in each group. ^*a*^*p* < 0.05 vs. sham group; ^*b*^*p* < 0.05 vs. CCI group; ^*c*^*p* < 0.05 vs. NSC group.

To investigate the ultrastructural changes of the sciatic nerve, we observed the changes in the myelin sheath of the injured sciatic nerve through transmission electron microscope. In the sham group, the structure of the myelin sheath of the sciatic nerve was uniform, regular, and clear. Myelin sheath axons were intact, nerve filaments, microtubules, and mitochondria were normal. However, in the CCI groups, the sciatic nerve fibers had the myelinated lamina separated, and disintegrated fragments could be seen. Part of the myelin sheath had segmental demyelination, axon atrophy and swelling, and visible telangiectasia and hyperemia. After NSC and MC-NSC transplantation, the myelin sheath of the sciatic nerve was relatively intact, and some demyelination changes could be seen, and there were visible axon growth and inflammation reduction. Compared with the NSC group, in the MC-NSCs group, the sciatic nerve had more myelinated fibers, the thickness of the myelin sheath was more uniform, and the morphology of the nerve filaments and mitochondria were normal (Figures 2A,B). Moreover, MBP (myelin basic protein) is the main protein of the myelin sheath. Therefore, we detected the changes in the fluorescence intensity of MBP in the myelin sheath of the sciatic nerve in each group of rats. The results showed that, compared with the sham group, the fluorescence intensity of MBP in the myelin sheath of the sciatic nerve in the CCI group was reduced, but compared with the CCI group, the fluorescence intensity of MBP in the myelin sheath of the NSCs and MC-NSCs groups was increased. In addition, compared with the NSC group, the sciatic nerve myelin staining intensity of the MC-NSCs group increased (Figure 2C). These data indicate that MC-NSCs transplantation has a better effect of repairing nerve damage.

### Detection Results of Mechanical Withdrawal Threshold of Rats

**FIGURE 3:**
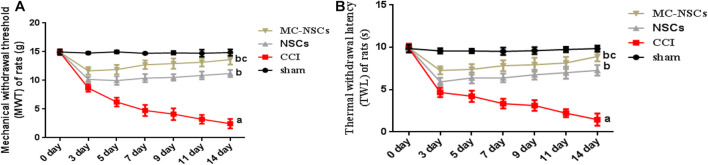
Behavioral methods were used to detect the mechanical withdrawal threshold (MWT) and thermal withdrawal latency (TWL) of rats in each group. **(A,B)** The MWT and TWL of rats from high to low was found in the sham, MC-NSC, NSC, and CCI groups (all *p*-values < 0.05), while compared with the NSCs group, the MWT and TWL of rats in the MC-NSC group were increased (*p* < 0.05). Data were expressed as the mean ± SD (*n* = 6), ^*a*^*p* < 0.05 vs. sham group; ^*b*^*p* < 0.05 vs. CCI group; ^*c*^*p* < 0.05 vs. NSC group.

To investigate the effect of NSC and MC-NSC transplantation on pain in rats, on days 0, 3, 5, 7, 9, 11, and 14 after operation, behavioral methods were used to measure the MWT and TWL of rats. The results showed that the MWT and TWL of the rats in the CCI group were significantly lower than those in the sham group (*p* < 0.05). The MWT and TWL of the rats in the NSCs and MC-NSCs groups were higher than those in the CCI group (*p* < 0.05). While compared with the NSC group, the MWT and TWL of the rats in the MC-NSC group were increased (*p* < 0.05) (Figures 3A,B). These data suggest that, with the increase in the time after NSC and MC-NSC transplantation, their pain-suppressing effect also increased. MC-NSC transplantation is better than NSC transplantation in suppressing pain.

### Detection Results of P2 × 4R Protein Expression

**FIGURE 4:**
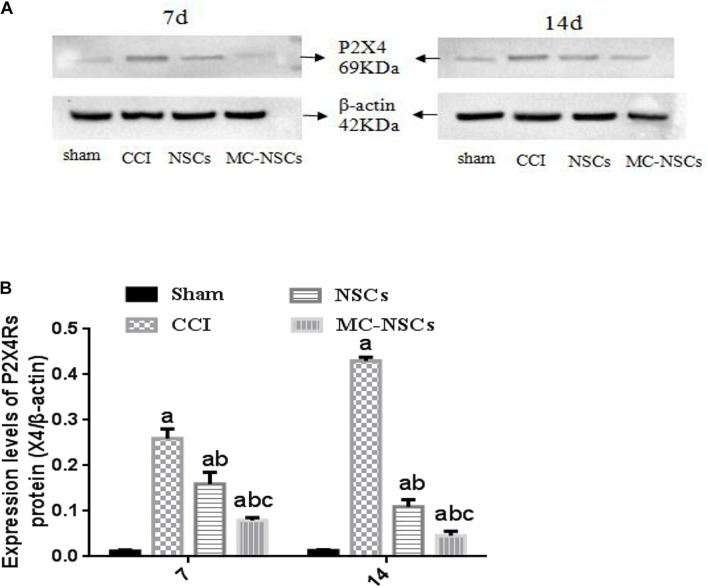
**(A)** Western blotting was performed to measure the expression of P2 × 4R protein in the L4–5 spinal cord segment tissues in each group. **(B)** The expression levels of P2 × 4R protein from high to low were found in the CCI, NSC, MC-NSC, and sham groups (all *p*-values < 0.05). Moreover, in the MC-NSC group, the expression of P2 × 4R protein was decreased on day 14 than that on day 7 (*p* < 0.05). Data were expressed as the mean ± SD of three replicates (*n* = 6). ^*a*^*p* < 0.05 vs. sham group; ^*b*^*p* < 0.05 vs. CCI group; ^*c*^*p* < 0.05 vs. NSC group.

To investigate the expression of P2 × 4R protein in the spinal cord tissue of each group of rats, on days 7 and 14 after operation, we used Western blotting to measure the expression level of P2 × 4R protein. The results showed that the integrated optical density (IOD) of P2 × 4Rs in the CCI group was significantly higher than those in the sham group (*p* < 0.05). The IOD of P2 × 4Rs in the NSC and MC-NSC groups were lower than those in the CCI group (*p* < 0.05). Moreover, compared with the NSC group, the IOD of P2 × 4Rs in the MC-NSC group was decreased (*p* < 0.05). Furthermore, we also investigated the changes in P2 × 4Rs expression over time after MC-NSCs transplantation. The results showed that, compared with the MC-NSCs group on day 7 after operation, the IOD of P2 × 4Rs in the MC-NSCs group was decreased on day 14 after operation (Figures 4A,B). These data indicate that MC-NSC transplantation can better reduce the overexpression of P2 × 4Rs.

### Results of Detection of P2 × 4R-Positive Cells

**FIGURE 5:**
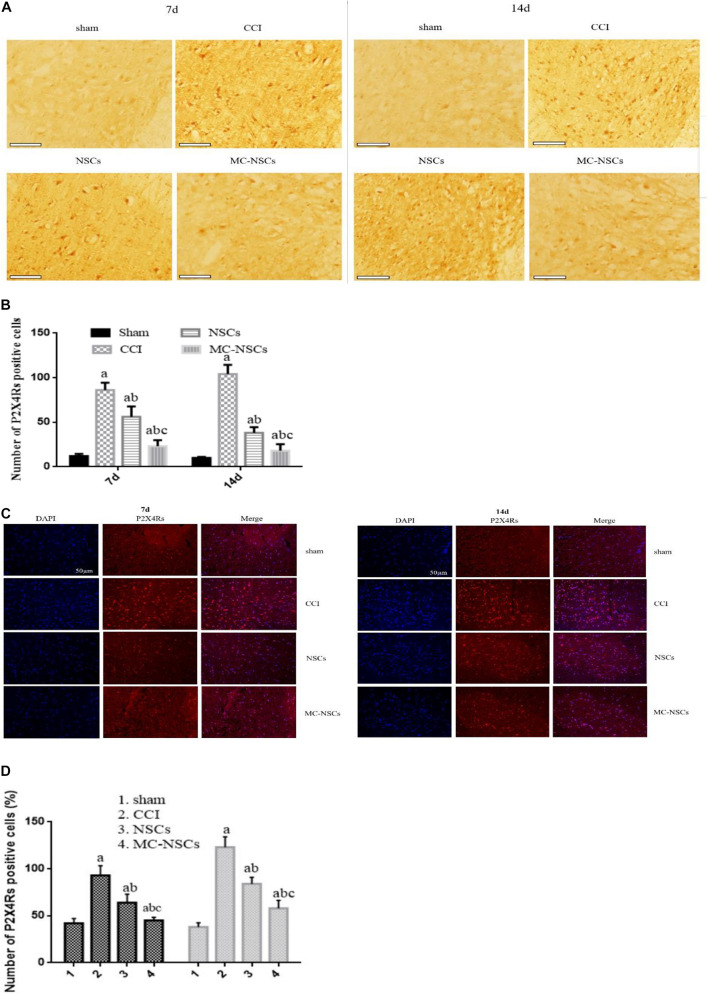
Expression of P2 × 4R-positive cells in the L4–5 spinal cord segment. **(A,B)** *In situ* hybridization was performed to detect the staining of P2 × 4R-positive cells. Bar = 100 μm. **(C,D)** Immunofluorescence was used to detect the expression of P2 × 4Rs in the spinal cord tissue of each group of rats. Data were expressed as the mean ± SD of three replicates (*n* = 6). ^*a*^*p* < 0.05 vs. sham group; ^*b*^*p* < 0.05 vs. CCI group; ^*c*^*p* < 0.05 vs. NSC group.

At 7 and 14 days after operation, we measured the number of P2 × 4R-positive cells by using *in situ* hybridization. Figure 5A shows that P2 × 4R-positive cells were stained brown or brownish yellow, and the staining of P2 × 4R-positive cells from deep to shallow was found in the CCI, NSC, MC-NSC, and sham groups. Interestingly, compared with the NSC group, the staining of P2 × 4R-positive cells in the MC-NSC group was lighter. Moreover, the number of P2 × 4R-positive cells from high to low was seen in the CCI, NSC, MC-NSC, and sham groups. Importantly, compared with the NSC group, the number of P2 × 4R-positive cells in the MC-NSC group was decreased (*p* < 0.05) (Figure 5B). Additionally, immunofluorescence was performed to detect the expression level of P2 × 4Rs in the spinal cord tissue of each group of rats, and similar results were obtained (Figures 5B–D). The above experimental data confirm that MC-NSCs transplantation can better reduce the overexpression of P2 × 4Rs.

### Results of P2 × 4R mRNA Expression in the Spinal Cord of Rats

**FIGURE 6:**
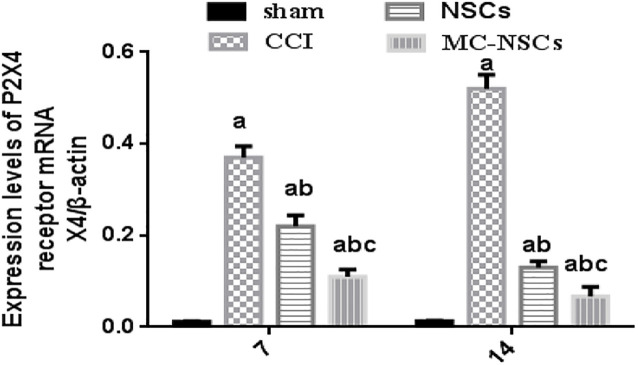
Results of P2 × 4R mRNA expression in the L4–5 spinal cord by using RT-qPCR. The expression level of P2 × 4R mRNA from high to low was found in the CCI, NSC, MC-NSC, and sham groups. Data were expressed as the mean ± SD of three replicates (*n* = 6). ^*a*^*p* < 0.05 vs. sham group; ^*b*^*p* < 0.05 vs. CCI group; ^*c*^*p* < 0.05 vs. NSC group.

On days 7 and 14 after surgery, we used RT-qPCR to measure the expression of P2 × 4R mRNA in the L4–5 spinal cord of rats. The results showed that the expression of P2 × 4R mRNA in the CCI group was higher than those of the sham group (*p* < 0.05). The expression of P2 × 4R mRNA in the NSC and MC-NSC groups were decreased than those of the CCI group (*p* < 0.05). However, compared with the NSC group, the expression of P2 × 4R mRNA in the MC-NSC group was decreased (*p* < 0.05). Moreover, the results also showed that the expression of P2 × 4R mRNA in the MC-NSCs group was decreased on day 14 after surgery than those in the MC-NSCs group on day 7 after surgery (*p* < 0.05) (Figure 6). These data indicate that MC-NSCs transplantation can better reduce the overexpression of P2 × 4Rs.

### Microencapsulated Neural Stem Cell Transplantation Decreases the Levels of IL-1β and TNF-a

**FIGURE 7:**
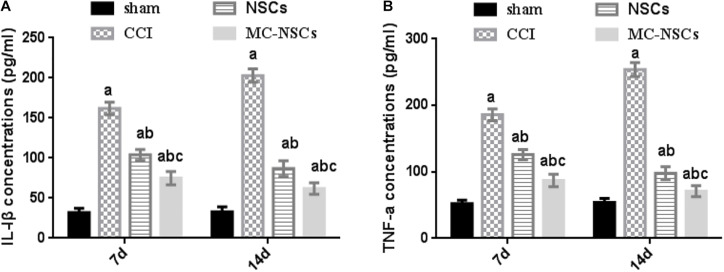
Results of IL-1β and TNF-a concentrations in serum by ELISA. **(A,B)** The concentrations of IL-1β and TNF-a in serum of rats from high to low were found in the CCI, NSCs, MC-NSCs, and sham groups. Compared with the NSCs group, the concentrations of IL-1β and TNF-a in the MC-NSCs group were reduced (*p* < 0.05). Data were expressed as the mean ± SD of three replicates (*n* = 6). ^*a*^*p* < 0.05 vs. sham group; ^*b*^*p* < 0.05 vs. CCI group; ^*c*^*p* < 0.05 vs. NSC group.

IL-1β and TNF-a are important inflammatory cytokines. Studies have shown that nerve injury can lead to an increase in the level of IL-1β and TNF-a, while reducing their release can reduce the inflammatory response of the nerve, which is beneficial to the repair of nerve injury (Huang et al., 2020; Wang et al., 2020). Therefore, we investigated the changes in IL-1β and TNF-a in rat serum after NSC and MC-NSC transplantation. The results showed that the concentrations of TNF-a and IL-1β in the serum of rats were significantly increased in the CCI group (*p* < 0.05). Moreover, compared with the CCI group on day 7 after surgery, the concentrations of TNF-a and IL-1β were higher in the CCI group on day 14 after surgery (*p* < 0.05). However, after NSC and MC-NSC transplantation, the concentrations of TNF-a and IL-1β were decreased (*p* < 0.05), while compared with the NSC group, the concentrations of TNF-a and IL-1β were decreased in the MC-NSC group (*p* < 0.05). Furthermore, after the MC-NSC transplantation, the levels of TNF-a and IL-1β were decreased on day 14 after surgery than those on day 7 after surgery (*p* < 0.05) (Figures 7A,B). These data indicate that MC-NSC transplantation better reduced the release of TNF-a and IL-1β.

### Microencapsulated Neural Stem Cell Transplantation Reduces the Expression of NF-kB p-p65 Protein

**FIGURE 8:**
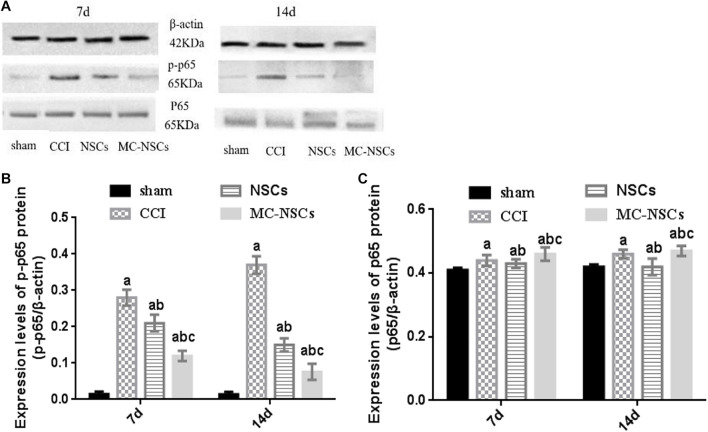
Results of p-p65 and p65 protein expression. **(A)** Western blotting was performed to measure the expression of p65 and p-p65 protein. **(B,C)** The integrated optical density (IOD) of p-p65 from high to low was found in the CCI, NSCs, MC-NSC, and sham groups. Compared with the NSCs group, the IOD of p-p65 in the MC-NSCs group was decreased *(p* < 0.05). However, there was no significant change in the IOD of total p65 in each group *(p* > 0.05). Data were expressed as the mean ± SD of three replicates (*n* = 6). ^*a*^*p* < 0.05 vs. sham group; ^*b*^*p* < 0.05 vs. CCI group; ^*c*^*p* < 0.05 vs. NSC group.

NF-kB is an important signaling pathway, which is involved in the development of nerve damage and inflammation (Peng et al., 2020). Therefore, we investigated whether MC-NSC transplantation has an effect on the expression of p-p65 (phosphorylated nuclear factor/K gene-binding nuclear factor p65). The results showed that, in the CCI group, the expression of p-p65 protein was higher than those in the other groups (*p* < 0.05). However, after NSC and MC-NSC transplantation, the expression of p-p65 protein was decreased (*p* < 0.05). Compared with the NSC transplantation, MC-NSC transplantation could better reduce the expression of p-p65 protein (*p* < 0.05). However, the total protein expression of p65 in each group remained unchanged (*p* > 0.05) (Figures 8A–C).

## Discussion

NPP is a common clinical symptom, mostly due to direct or indirect damage to the nervous system, leading to motor and sensory dysfunction, seriously affecting the psychosomatic health of patients (Gilron et al., 2015). Currently, the role of P2X receptor is widely studied in the pathological mechanism of NPP. P2X is dependent on the ATP ion channel receptor and plays a key role in the development of nerve injury and pain (Kato et al., 2017). P2 × 4 receptors are important members of the P2X family and play a key role in the development of pain (Xiaodi et al., 2010; Deng et al., 2018). After the nervous system is subjected to noxious stimuli, ATP is released in large quantities, activates the P2 × 4 receptors, enhances sensory information transmission, sensitizes the central nervous system, and induces pain. On the contrary, reducing the expression levels of P2 × 4 receptors can inhibit pain (Wang et al., 2019). The results of this study have shown that high expression of P2 × 4 receptors in the spinal cord can induce hyperalgesia in rats, which is consistent with previous related studies. With the exploration and research of NPP treatment methods, the concept of cell transplantation was proposed (Lindvall, 2015). In recent years, cell transplantation has become a prospective treatment method for repairing damaged nerves and relieving pain. In our previous study, we transplanted olfactory ensheathing cells into the site of the injured sciatic nerve and found that it could reduce the overexpression of P2 × 4 receptors and inhibit pain (Zhang et al., 2019b). In order to further explore the treatment of NPP, we took advantage of the unique biological characteristics of NSCs. For example, NSCs can self-renew and maintain a stable number, differentiate into all tissues and cells in the nervous system, secrete more neurotrophic factors, inhibit the formation of glial scars and cavities, promote axonal regeneration, and repair injured nerves (Itakura et al., 2017; Hu et al., 2019). However, transplantation of heterologous cells into the host can cause immune rejection and damage the transplanted cells. Microcapsules are biological lipid translucent membranes that allow some small molecular substances to pass through (such as oxygen and amino acids), hinder macromolecular substances (such as inflammatory factors), exert the role of immune isolation, and reduce damage factors to attack transplanted cells (Saenz del Burgo et al., 2017; Karpov et al., 2019).

In this study, the unique biological characteristics of NSCs and microcapsules were used, and an animal model of chronic sciatic nerve compression injury was established. NSCs and MC-NSCs were transplanted into the site of sciatic nerve injury to explore the therapeutic effect of inhibiting pain and compared the differences between them. Our results found that, after sciatic nerve injury, sciatic nerve myelin sheath was broken, demyelination changes could be seen, there was axon atrophy, and the fluorescence intensity of myelin MBP weakened. The MWT and TWL of rats were significantly reduced, the expression levels of P2 × 4Rs and p-p65 were increased, and the concentrations of TNF-a and IL-1β were significantly increased. After the transplantation of NSCs and MC-NSCs, more nerve fibers could be seen, myelin sheath was intact, and axon regeneration could be seen. The fluorescence intensity of myelin MBP increased. The MWT and TWL of rats were significantly increased, and the expression levels of P2 × 4Rs and p-p65 were significantly reduced. However, compared with the transplantation of NSCs, MC-NSCs could better reduce the expression level of P2 × 4Rs and p-p65, decrease the releases of TNF-a and IL-1β, and relieve pain. Moreover, with the increase in the time of NSC and MC-NSC transplantation, the effect of suppressing pain increased accordingly. Therefore, through the results of this study, our conclusion is that MC-NSCs can better inhibit pain through reducing the expression of P2 × 4Rs and inhibiting the activation of NF-kB signaling, decrease the release of inflammatory factors IL-1β and TNF-α, and alleviate pain, and the therapeutic effects were better than those of NSC transplantation. The mechanism may include the following aspects: (1) NSCs secrete neurotrophic factors, change the microenvironment around nerve damage, repair damaged nerves, and promote axon regeneration (Wang et al., 2018; Zhou et al., 2018). (2) NSCs can inhibit nerve demyelination, reduce the expression of P2 × 4Rs in the spinal cord, decrease sensory information transmission, and relieve pain (Levi et al., 2018). (3) Microcapsules have the function of immune isolation barrier, protect the attack of inflammatory factors on transplanted cells, help transplanted cells play a role in repairing damaged nerves, and better reduce the expression level of P2 × 4Rs, thereby inhibiting pain (Zhang et al., 2018a, 2019a).

Through this experimental study, NSCs and MC-NSCs can inhibit P2 × 4R overexpression-mediated NPP. However, we also found some deficiencies, such as the optimal time for cell transplantation and the optimal dose of transplanted cells. These related issues need to be further explored and solved in the future. These experimental data indicate that MC-NSCs have a better therapeutic effect for pain relief, which becomes another promising method for the treatment of NPP.

## Data Availability Statement

The original contributions presented in the study are included in the article/supplementary material, further inquiries can be directed to the corresponding author/s.

## Ethics Statement

All protocols were approved by the Animal Care and Ethics Committee, China.

## Author Contributions

W-JZ carried out the study of the experiment and drafted the manuscript. CL, CH, and S-CL completed the behavioral method testing and collected the data. H-LL helped revise this manuscript. All authors read and approved the final manuscript.

## Conflict of Interest

The authors declare that the research was conducted in the absence of any commercial or financial relationships that could be construed as a potential conflict of interest.

## Publisher’s Note

All claims expressed in this article are solely those of the authors and do not necessarily represent those of their affiliated organizations, or those of the publisher, the editors and the reviewers. Any product that may be evaluated in this article, or claim that may be made by its manufacturer, is not guaranteed or endorsed by the publisher.

## Abbreviations

CCI: chronic sciatic nerve compression injury
MWT: mechanical withdrawal threshold
NPP: neuropathic pain
SSC: (NaCl, sodium citrate, ddH_2_O_2_) buffer solution
NSCs: neural stem cells
MC-NSCs: microencapsulated neural stem cells
P2 × 4Rs: P2 × 4 receptors
PBS: phosphate-buffered saline
SD: Sprague–Dawley
SABC: streptavidin–biotin complex
TWL: thermal withdrawal latency.
